# lncRNA MEG3 inhibits the growth of hepatocellular carcinoma cells by sponging miR-9-5p to upregulate SOX11

**DOI:** 10.1590/1414-431X20198631

**Published:** 2019-09-16

**Authors:** Zhi Liu, Jian Yu Chen, Yang Zhong, Liang Xie, Jian Shui Li

**Affiliations:** 1 Department of Hepatobiliary Surgery, Affiliated Hospital of North Sichuan Medical College, Nanchong, China; 2 Institute of Hepatobiliary, Pancreatic and Intestinal Disease, North Sichuan Medical College, Nanchong, China

**Keywords:** Hepatocellular carcinoma, MEG3, miR-9-5p, SOX11, lncRNA

## Abstract

The long non-coding RNA (lncRNA) maternally expressed gene 3 (MEG3), a tumor suppressor, is critical for the carcinogenesis and progression of different cancers, including hepatocellular carcinoma (HCC). To date, the roles of lncRNA MEG3 in HCC are not well illustrated. Therefore, this study used western blot and qRT-PCR to evaluate the expression of MEG3, miR-9-5p, and Sex determining Region Y-related HMG-box 11 (SOX11) in HCC tissues and cell lines. RNA pull-down and luciferase reporter assay were used to evaluate these molecular interactions. 3-(4,5-dimethylthiazol-2-yl)-2,5-diphenyltetrazolium bromide and flow cytometry detected the viability and apoptosis of HCC cells, respectively. The results showed that MEG3 and SOX11 were poorly expressed but miR-9-5p was highly expressed in HCC. The expression levels of these molecules suggested a negative correlation between MEG3 and miR-9-5p and a positive correlation with SOX11, confirmed by Pearson's correlation analysis and biology experiments. Furthermore, MEG3 could combine with miR-9-5p, and SOX11 was a direct target of miR-9-5p. Moreover, MEG3 over-expression promoted cell apoptosis and growth inhibition in HCC cells through sponging miR-9-5p to up-regulate SOX11. Therefore, the interactions among MEG3, miR-9-5p, and SOX11 might offer a novel insight for understanding HCC pathogeny and provide potential diagnostic markers and therapeutic targets for HCC.

## Introduction

Hepatocellular carcinoma (HCC) is the third leading cause of cancer-related deaths globally, accounting for approximately 90% of liver cancer (1,2). There are a variety of risk factors for HCC, mainly including obesity, smoking, alcohol consumption, liver cirrhosis, and chronic infection with hepatitis B and hepatitis C (3). Surgical resection, orthotopic liver transplantation, transarterial chemoembolization, radiofrequency ablation, and systemic chemotherapies are available treatments for HCC patients (4). Nevertheless, metastasis and recurrence still occur in varying degrees (4,5). New options to improve HCC patient survival rate are still badly needed, and exploring the molecular mechanisms of HCC may offer new insights into HCC treatment.

Numerous reports suggest that the gene regulatory networks are relevant for the development of cancer (6). Long non-coding RNAs (lncRNAs; containing >200 nucleotides) are known as key regulators for various cellular processes at transcriptional, posttranscriptional, and epigenetic levels (7,8). The regulation of lncRNA for biological processes has diverse molecular mechanisms such as RNA-RNA, RNA-protein, and RNA-DNA interactions (9). For instance, lncRNA maternally expressed gene 3 (MEG3) has been reported to function as a tumor suppressor in various cancers such as hemangioma (10), osteosarcoma (11), and thyroid carcinoma (12), and it can serve as a ceRNA for miR-9-5p to mediate the progression of esophageal cancer (13) and prostate cancer (14). Although lncRNA MEG3 has been demonstrated to be down-regulated in HCC (15,16), the roles of the interaction between MEG3 and miR-9-5p in HCC have not been reported.

Sex determining region Y-related HMG-box 11 (SOX11) is a type of structurally-related transcription factor and belongs to group C of SOX family (17,18). SOX11 is critical for regulating organ development, particularly in the nervous system (19,20). Accumulating evidence shows that SOX11 is involved in the biological processes of many human cancers (21), whereas the impact of SOX11 on the progression of different cancers is still controversial and seems to be determined by cancer types (18,22,23). It has been reported that SOX11 is poorly expressed in HCC tissues (24) and yet, the roles of SOX11 on HCC are not well illustrated.

In the present study, we determined the expression of MEG3, miR-9-5p, and SOX11 in HCC tissues, and explored their interactions in HCC. Furthermore, we detected the effect of these molecules on HCC cell growth and apoptosis.

## Material and Methods

### Human specimens

A total of 30 pairs of hepatocellular carcinoma (HCC) tissues and the corresponding adjacent normal tissues were obtained from HCC patients undergoing surgery at the Affiliated Hospital of North Sichuan Medical College (China). None of the patients had received chemotherapy or radiotherapy prior to surgery. Before surgery, written informed consent was collected from HCC patients. This work was approved by the Affiliated Hospital of North Sichuan Medical College's Ethics Committee. The clinical characteristics of the HCC patients are summarized in Table 1.

**Table 1.** Clinical characteristics of the hepatocellular carcinoma patients.

Characteristics	Number of patients	High MEG3 expression (%)	Low MEG3 expression (%)	P value
Gender				0.526
Male	23	10 (43.48)	13 (56.52)	
Female	7	4 (57.14)	3 (42.86)	
Age (years)				0.654
<55	18	9 (50.00)	9 (50.00)	
≥55	12	5 (41.67)	7 (58.33)	
Tumor size (cm)				0.464
<5	15	8 (53.33)	7 (46.67)	
≥5	15	6 (40.00)	9 (60.00)	
Serum AFP (ng/mL)				0.818
<20	7	3 (42.86)	4 (57.14)	
≥20	23	11 (47.83)	12 (52.17)	
HBsAg				0.873
Negative	9	4 (44.44)	5 (55.56)	
Positive	21	10 (47.62)	11 (52.38)	
Liver cirrhosis				0.919
Absence	11	5 (45.45)	6 (54.55)	
Presence	19	9 (47.37)	10 (52.63)	
Histological differentiation				0.727
Well	4	2 (50.00)	2 (50.00)	
Moderate	13	5 (38.46)	8 (61.54)	
Poor	13	7 (53.85)	6 (46.15)	
TNM stage				0.028
I + II	15	10 (66.67)	5 (33.33)	
III + IV	15	4 (26.67)	11 (73.33)	
Metastasis				<0.001
No	15	12 (80.00)	3 (20.00)	
Yes	15	2 (13.33)	13 (86.67)	

Data are reported as number and percentage. Chi-squared test was used to compare groups.

### Cell culture and transfection

Human embryonic kidney cell line (293T) and Human HCC cell lines (SK-HEP-1 and Huh7) were purchased from Institute of Biochemistry and Cell Biology (China), and cultured in complete DMEM medium (Gibco, USA). The plasmid for mediating MEG3 overexpression (pcNDA-MEG3), the control plasmid (pcNDA-Vector), siRNAs for knockdown of MEG3 (MEG3 siRNA1 and MEG3 siRNA2), and the control siRNA (NC siRNA) were obtained from GenePharma (China). After adding to twelve-well plates, SK-HEP-1 and Huh7 cells were transfected with pcNDA-MEG3, pcNDA-Vector, MEG3 siRNA1, MEG3 siRNA2, or NC siRNA for 24–48 h using lipofectamine 3000 according to the manufacturer's protocol (Invitrogen, USA). The transfected cells were harvested for the following experiments.

### Quantitative real-time PCR (qRT-PCR)

Total RNA was isolated from each tumor sample and HCC cells by TRIzol reagent (Invitrogen), and then reverse transcribed with the First Strand cDNA synthesis kit (New England Biolabs (Beijing) LTD., China). We performed amplifications with a SYBR Green PCR kit (Applied Biological Materials, Canada) according to the manufacturer's instructions on Applied Biosystems 7500 Real-Time PCR System (Applied Biosystems, USA). The expression of RNA was normalized against GAPDH using the 2^-△△Ct^ method. The PCR primers used are shown in Table 2. Three separate experiments were performed.

**Table 2.** Primer sequence.

Primer	Sequence (5′-3′)
MEG3 forward	CTGCCCATCTACACCTCACG
MEG3 reverse	CTCTCCGCCGTCTGCGCTAGGGGCT
miR-9-5p forward	GTGCAGGGTCCGAGGT
miR-9-5p reverse	GCGCTCTTTGGTTATCTAGC
SOX11 forward	GGTGGATAAGGATTTGGATTCG
SOX11 reverse	GCTCCGGCGTGCAGTAGT
GAPDH forward	CAACGAATTTGGCTACAGCA
GAPDH reverse	AGGGGTCTACATGGCAACTG

### Western blot

The tissues and cell lines of HCC were lysed by an ice-cold RIPA lysis buffer (Beyotime, China) containing a protease inhibitor cocktail (Sigma-Aldrich, USA). The lysates were quantified using BCA methods and equally loaded on SDS-PAGE gels and electroblotted onto polyvinylidene fluoride (PVDF) membranes. After blocking the membranes with 5% skim milk powder, we incubated the membranes with the primary antibodies (1:1000 dilution) against SOX11 (Epitomics, USA), Bcl-2 (Cell Signaling Technology, USA), cleaved caspase-3 (Cell Signaling Technology), cleaved PARP (Cell Signaling Technology), and GAPDH (Cell Signaling Technology) overnight at 4°C. Next, we cultured the membranes with HRP-conjugated secondary antibodies for 2 h at 25°C. Finally, the membranes were visualized using ECL-PLUS/Kit (GE Healthcare, USA) and the protein was quantified using ImageJ software (National Institutes of Health, USA).

### RNA pull-down assay

RNA pull-down assay was used to detect whether lncRNA MEG3 is relevant for miR-9-5p. In this assay, the biotin-labeled MEG3, as a probe, or random pull-down probe sequence, as negative control (NC), were reversely transcribed via Biotin RNA labeling mix (Roche Diagnostics, USA) and T7 RNA polymerase (Roche, Switzerland). Subsequently, the samples were processed by RNase-free DNase I (Roche) and then purified using the RNeasy Mini Kit (Qiagen, USA). Huh7 and SK-HEP-1 cell lines were lysed in RIPA buffer for 30 min and the lysates were mixed with biotin-labeled MEG3 followed by incubating for 1 h at 4°C. Subsequently, the reaction mixture was incubated with streptavidin agarose beads (Life Technologies, USA) for 1 h at room temperature. qRT-PCR was utilized to measure the co-precipitated RNAs.

### Luciferase reporter assay

3′-untranslated regions (UTRs) of SOX11 including the predicted wild-type (WT) binding sites of miR-9-5p or mutant binding sites (Mut) were inserted into a luciferase reporter vector (Promega, USA), designated as WT-SOX11 and Mut-SOX11. HCC cells were collected 48 h after co-transfection of miR-9-5p mimics or pcNDA-MEG3 along with luciferase reporter vectors. Then, the relative luciferase activity of the cells was analyzed via the dual-luciferase reporter system (Promega).

### Cell viability assay

In the present study, we applied 3-(4,5-dimethylthiazol-2-yl)-2,5-diphenyltetrazolium bromide (MTT) assay to evaluate the viability of HCC cells. Cells transfected with MEG3 overexpression plasmid (MEG3), MEG3 + miR-9-5p mimics, or siSOX11 were cultured with complete DMEM medium in 96-well plates for 24, 48, and 72 h, respectively. Next, the cells were treated with MTT solution (0.5%, dissolved in DMSO, Beyotime) and incubated for another 4 h at 37°C. The absorbance of the samples was assessed at 490 nm by a reader (EL 800 Universal Microplate reader, BioTek, USA).

### Flow cytometry

HCC cells were transfected with MEG3 overexpression plasmid (MEG3), MEG3 + miR-9-5p mimics, or MEG3 + siSOX11 for 48 h. These treated cells were harvested and re-suspended in binding buffer. Next, annexin V-FITC and propidium iodide (PI) were utilized to stain these cells. Then, flow cytometry (FACSCanto II, BD Biosciences, USA) was utilized to determine the apoptotic cells.

### Statistical analysis

Data are reported as means±SD and were analyzed by GraphPad Prism 7.0 software (GraphPad Software Inc., USA). The significant difference between groups was evaluated using Student's *t*-test for a single comparison or one-way analysis of variance (ANOVA) with Bonferroni *post-hoc* test for multiple comparisons. A value of P<0.05 was considered statistically significant.

## Results

### Expression of MEG3, miR-9-5p, and SOX11 in HCC tissues

**Figure 1.**
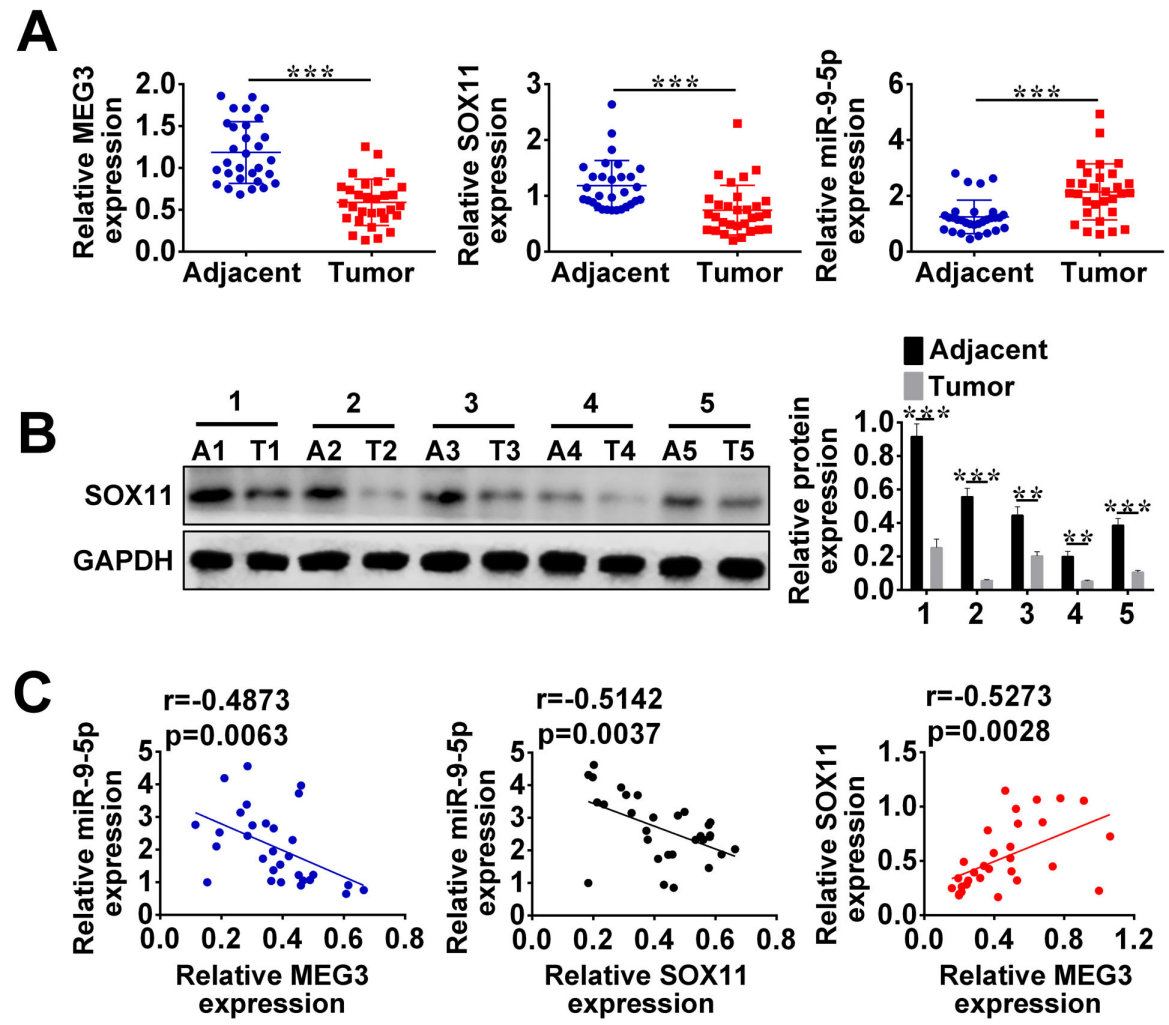
**A**, qRT-PCR detected the expression of MEG3, SOX11, and miR-9-5p in hepatocellular carcinoma tissues (HCC). **B**, Western blot was utilized to measure the protein expression of SOX11 in five random HCC tissues. **C**, Interactions among SOX11, miR-9-5p, and MEG3 were assessed by Pearson's correlation analysis. Data are reported as means±SD. **P<0.05, ***P<0.01, *vs* adjacent tissue (A, control group) (Student's *t*-test). T: Tumor tissue.

To determine the expression of MEG3, miR-9-5p, and SOX11 in HCC tissues, we analyzed their expressions using qRT-PCR. The results revealed that the expression levels of MEG3 and SOX11 were down-regulated but miR-9-5p was highly expressed in HCC tissues compared to the corresponding adjacent normal tissues (Figure 1A). SOX11 was poorly expressed in HCC tissues compared to the adjacent normal tissues, confirmed by western blot (Figure 1B). Furthermore, Pearson's correlation analysis indicated that lncRNA MEG3 had a negative correlation with miR-9-5p and displayed a positive correlation with SOX11 in HCC tissues. There was a negative correlation between SOX11 and miR-9-5p (Figure 1C).

### MEG3 served as a sponge for miR-9-5p in HCC cells

**Figure 2.**
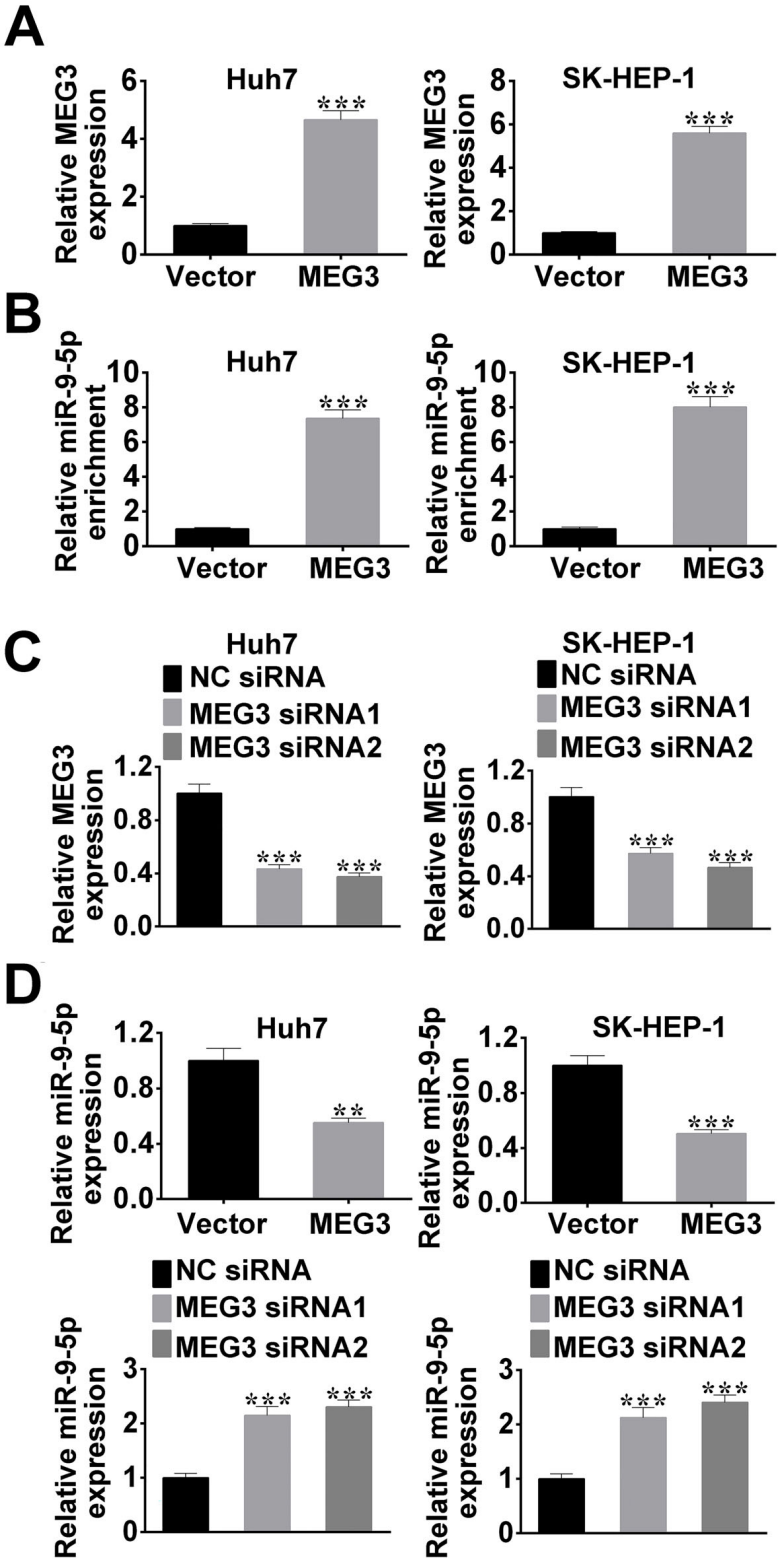
A, qRT-PCR detected the expression of MEG3 in hepatocellular carcinoma (HCC) cells transfected with pcNDA-MEG3 (MEG3) or control vector. **B**, RNA pull-down assay was used to determine the relative miR-9-5p enrichment in HCC cells, and the mRNA level was measured using qRT-PCR. **C**, qRT-PCR determined MEG3 expression in HCC cells transfected with MEG3 siRNA1, MEG3 siRNA2, or control siRNA (NC siRNA). **D**, Relative expression of mi-9-5p was assessed using qRT-PCR after HCC cells transfection with pcNDA-MEG3 (MEG3), control vector, MEG3 siRNA1, MEG3 siRNA2, or control siRNA (NC siRNA). Data are reported as means±SD. **P<0.05, ***P<0.01 *vs* control group (ANOVA).

The correlation between MEG3 and miR-9-5p was further explored in HCC cells. Their interaction was firstly determined by RNA pull-down assay and qRT-PCR. Compared with the control RNA, a significant enrichment of miR-9-5p in the MEG3 pulled-down pellet was observed (Figure 2A). Thus, MEG3 could directly combine with miR-9-5p.

As shown in Figure 2B and C, the expression of MEG3 was significantly up-regulated and down-regulated after HCC cells transfected with pcNDA-MEG3 (MEG3) and MEG3 siRNAs (MEG3 siRNA1 or MEG3 siRNA2), respectively.

The interaction between MEG3 and miR-9-5p was further assessed by qRT-PCR. Compared with the control group, miR-9-5p expression in HCC cells was decreased by the transfection of pcNDA-MEG3, while miR-9-5p expression in HCC cells was enhanced after MEG3 siRNAs transfection (Figure 2D). Therefore, MEG3 served as a sponge for miR-9-5p in HCC cells.

### Relationship among MEG3, miR-9-5p, and SOX11 in HCC cells

**Figure 3.**
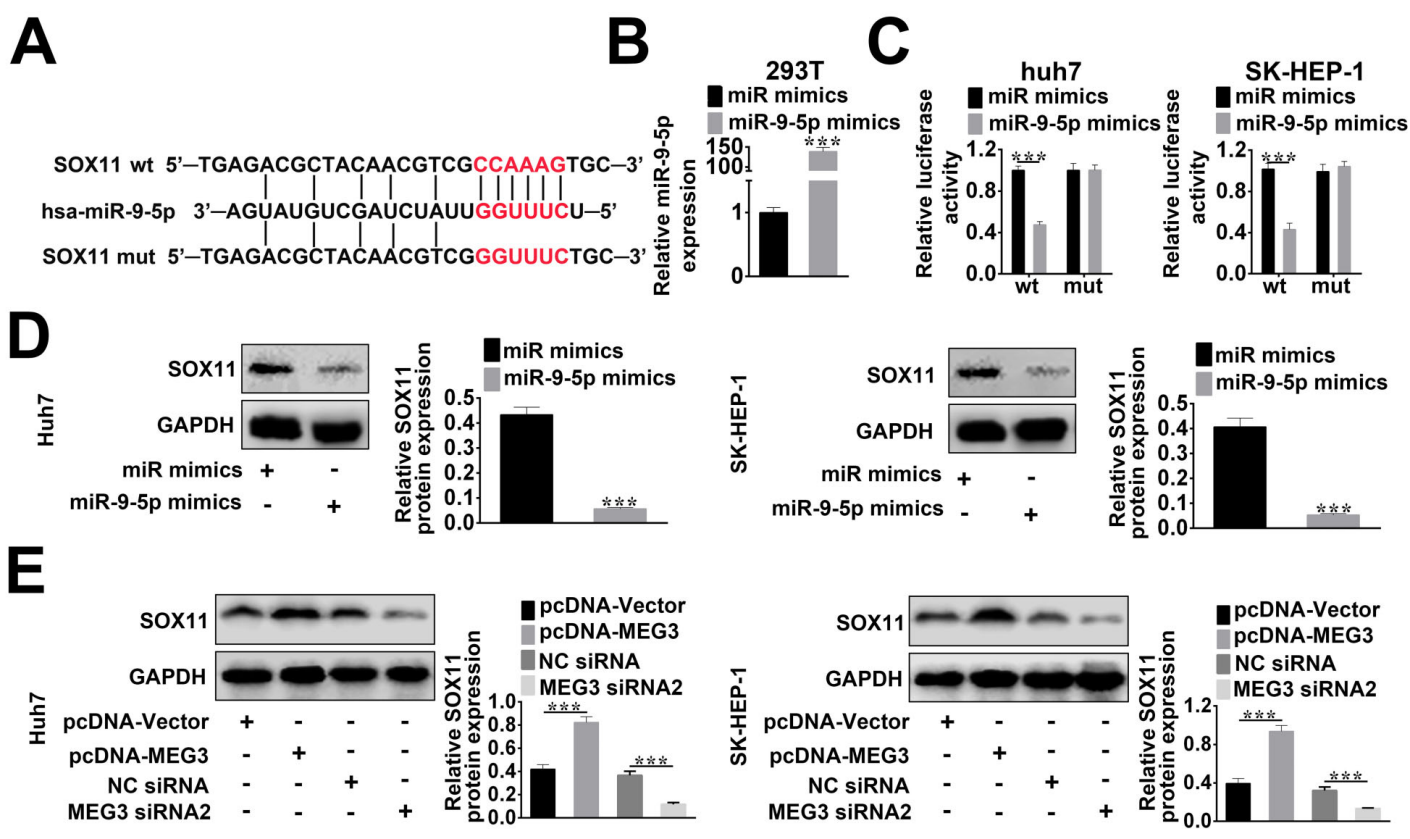
**A**, StarBase and mirBase software were applied to predict the targeting relationship between SOX11 and miR-9-5p. **B**, After hepatocellular carcinoma (HCC) cells were transfected with miR-9-5p mimics or control mimics (miR mimics) for 48 h, the miR-9-5p expression level was assessed using qRT-PCR. **C**, Luciferase reporter assay in HCC cell lines (huh7 and SK-HEP-1) was used to evaluate the relationship between SOX11 and miR-9-5p. **D**, Western blot detected the protein expression of SOX11 in HCC cells transfected with miR-9-5p mimics or control mimics (miR mimics). **E**, After HCC cells were transfected with pcNDA-MEG3 (MEG3), control vector, MEG3 siRNA2, or control siRNA (NC siRNA), western blot was used to determine the SOX11 expression. Data are reported as means±SD. ***P<0.01 *vs* control group (*t*-test).

StarBase (<http://starbase.sysu.edu.cn/starbase2/index.php>) and mirBase software (<http://www.mirbase.org>) were used to predict the targeting relationship between SOX11 and miR-9-5p (Figure 3A). As shown in Figure 3B, miR-9-5p expression in 293T cells was significantly up-regulated after miR-9-5p mimic transfection, and thus the miR-9-5p mimic transfection was effective. Additionally, the luciferase reporter assay suggested that HCC cell lines (huh7 and SK-HEP-1) co-transfected with miR-9-5p mimics and SOX11-WT showed a weakened luciferase activity in comparison to the control group (miR control mimics + SOX11-WT) (Figure 3C). Furthermore, western blot indicated that SOX11 expression was decreased by miR-9-5p mimic transfection (Figure 3D). Data suggested that SOX11 was a direct target of miR-9-5p. In addition, the negative correlation between SOX11 and miR-9-5p was consistent with results shown in Figure 1C.

The relationship between MEG3 and SOX11 in HCC cells was further validated by western blot. As shown in Figure 3E, pcDNA-MEG3 greatly increased the expression of SOX11, whereas MEG3 siRNA decreased SOX11 expression in comparison to the control group. Hence, MEG3 could enhance the expression of SOX11 and a positive correlation between MEG3 and SOX11 was consistent with results shown in Figure 1C.

### MEG3 regulated the growth of HCC cells via mediating miR-9-5p

**Figure 4.**
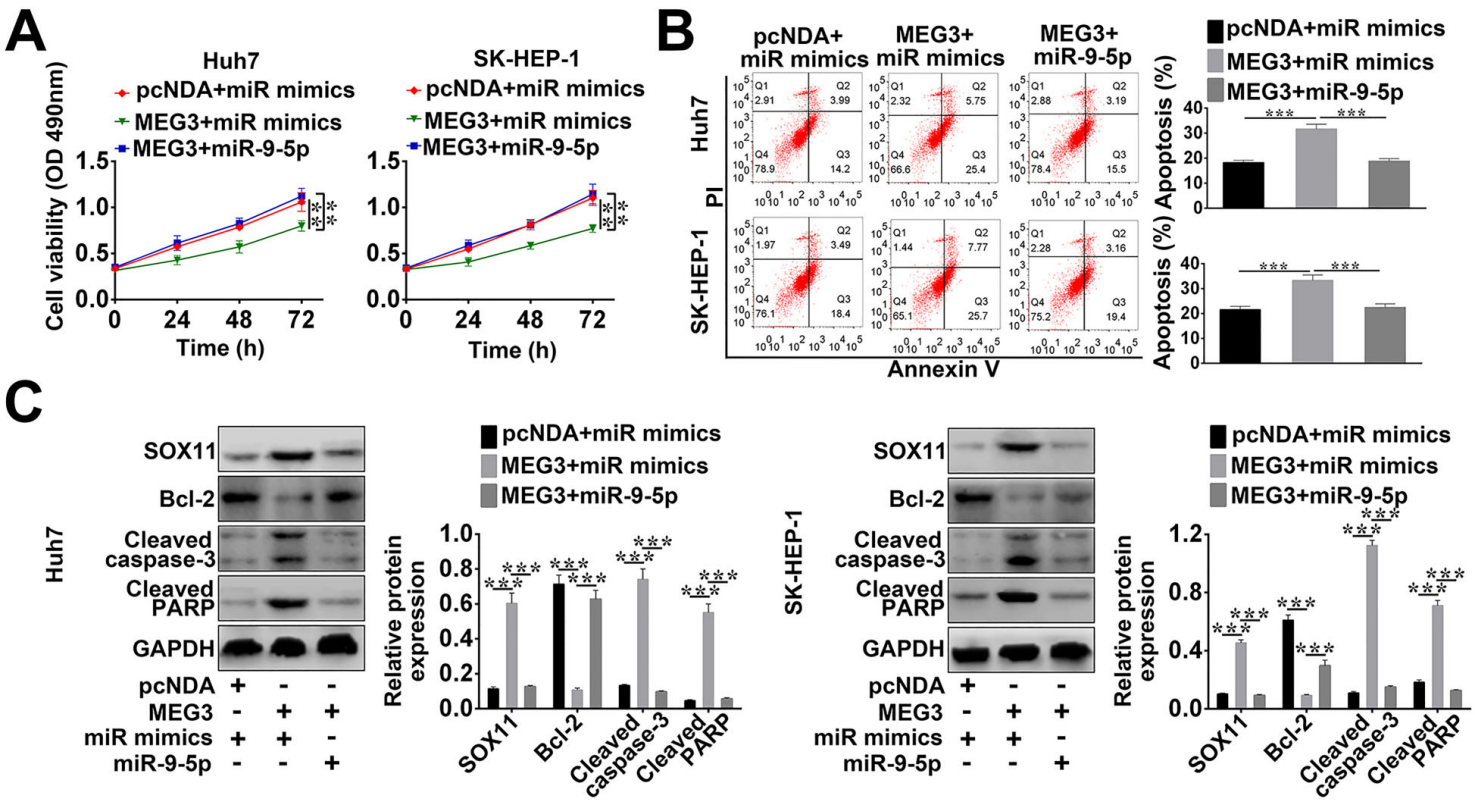
MEG3 regulated the growth of hepatocellular carcinoma (HCC) cells via mediating miR-9-5p. HCC cells were transfected with pcNDA-MEG3 (MEG3) + control mimics (miR mimics), pcNDA-MEG3 (MEG3) + miR-9-5p mimics, or control vector + control mimics (miR mimics), and then (**A**) the transfected cell viability was assessed using MTT. **B**, The transfected cell apoptosis was measured using flow cytometry. **C**, The protein expression levels of BCL-2, cleaved PARP, cleaved caspase-3, and SOX11 were measured by western blot. Data are reported as means±SD. **P<0.05, ***P<0.01 (ANOVA or *t*-test).

To detect the role of MEG3 on HCC cells, MTT, flow cytometry, and western blot were used in this study. MTT assay revealed that MEG3 over-expression induced by pcDNA-MEG3 transfection considerably inhibited the viability of HCC cells, while the cell viability was reversed by the introduction of miR-9-5p mimics (Figure 4A). Thus, miR-9-5p mimics could inverse the inhibition of HCC cell viability induced by MEG3 over-expression.

Flow cytometry analysis showed MEG3 over-expression (huh7 cell apoptotic rate, 31.15%; SK-HEP-1 cell apoptotic rate, 33.47%) significantly induced the apoptosis of HCC cell compared to MEG3 plasmid combined with miR-9-5p mimics transfection group (huh7 cell, 18.69%; SK-HEP-1cell, 22.56%) and the control group (pcDNA + miR mimics) (huh7 cell, 18.19%; SK-HEP-1cell, 21.89%). In addition, western blot demonstrated that the low expression of the anti-apoptosis protein Bcl-2 induced by MEG3 over-expression plasmid transfection was greatly changed by the transfection of miR-9-5p (Figure 4C). Furthermore, the expression of pro-apoptosis proteins cleaved PARP and cleaved caspase-3 was up-regulated by MEG3 over-expression plasmid transfection, whereas co-transfection with pcDNA-MEG3 and miR-9-5p considerably decreased their expression (Figure 4C). Collectively, MEG3 over-expression promoted the apoptosis of HCC cells, but this situation could be changed by miR-9-5p mimics introduction.

Additionally, the high expression of SOX11 in MEG3 over-expression group was remarkably decreased by the introduction of miR-9-5p mimics (Figure 4C). Therefore, MEG3 over-expression inhibited the viability of HCC cells, induced cell apoptosis, and enhanced SOX11 expression by mediating miR-9-5p.

### MEG3 regulated the growth of HCC cells via sponging miR-9-5p to regulate SOX11 expression

**Figure 5.**
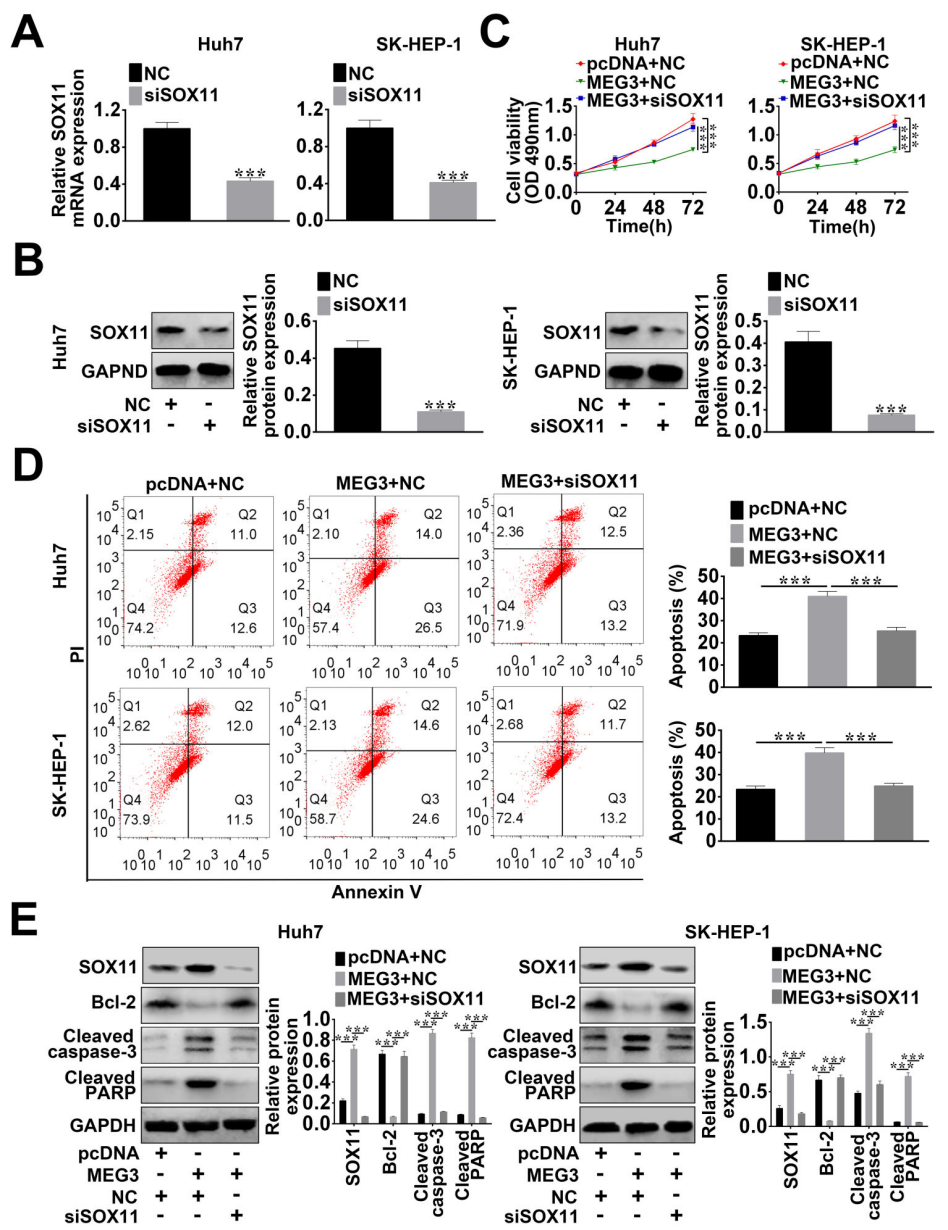
MEG3 regulated the growth of hepatocellular carcinoma (HCC) cells through sponging miR-9-5p to regulate SOX11 expression. **A**, mRNA and (**B**) protein expression of SOX11 were assessed using qRT-PCR and western blot, respectively. HCC cells were then transfected with pcNDA-MEG3 (MEG3) + siRNA control (NC), pcNDA-MEG3 (MEG3) + siSOX11, or control vector + siRNA control (NC). **C**, Viability of HCC cells was detected by MTT. **D**, Apoptosis rate of HCC cells was determined using flow cytometry. **E**, Protein expression levels of BCL-2, cleaved PARP, cleaved caspase-3, and SOX11 were measured using western blot. Data are reported as means±SD. ***P<0.01 (ANOVA or *t*-test).

On the one hand, it has been reported that SOX11 overexpression promotes apoptosis and growth inhibition in HCC cell (24); on the other, a negative correlation between MEG3 and miR-9-5p and a positive correlation with SOX11 have been confirmed. We speculated that the inhibition of MEG3 over-expression on HCC cells might be through sponging miR-9-5p to regulate SOX11 expression. In Figure 5A and B, the transfection of SOX11 siRNA (siSOX11) in HCC cells was effective, which was validated by qRT-PCT and western blot. As shown in Figure 5C, MEG3 over-expression inhibited cell viability and yet, siSOX11 introduction inversed the situation. Thus, SOX11 knockdown could improve the viability in HCC cells transfected with pcDNA-MEG3.

In addition, the apoptotic rates of Huh7 cells transfected with control plasmid, pcDNA-MEG3, or combined with siSOX11 were 23.6, 40.5, and 25.7%, respectively; the apoptotic rates of SK-HEP-1 cells transfected with control plasmid, pcDNA-MEG3, or combined with siSOX11 were 23.5, 39.2 and 24.9%, respectively (Figure 5D). Western blot further confirmed that the low expression of Bcl-2 and the high expression of SOX11, cleaved PARP, and cleaved caspase-3 induced by pcDNA-MEG3 transfection were changed via the introduction of siSOX11 (Figure 5E). Thus, MEG3 over-expression induced the apoptosis of HCC cells, which could be changed by knockdown of SOX11.

Collectively, MEG3 promoted apoptosis and growth inhibition in HCC cell through sponging miR-9-5p to up-regulate SOX11 expression.

## Discussion

This study observed the low expression of MEG3 and SOX11 and the high expression of miR-9-5p in HCC. A negative correlation between MEG3 and miR-9-5p and a positive correlation with SOX11 were demonstrated. MEG3 acted as a sponge of miR-9-5p, and SOX11 was a direct target of miR-9-5p. Moreover, MEG3 regulated the growth of HCC cells via sponging miR-9-5p to regulate SOX11 expression.

The molecular mechanism of HCC pathology needs to be explored, as HCC possesses a high mortality rate and lacks effective therapies (1,2). lncRNAs have been reported to be relevant with many cancer-related molecular mechanisms (25). lncRNA regulatory networks (e.g. RNA-RNA, RNA-protein, and RNA-DNA interactions) are critical for cellular processes (9). In the present study, we selected tumor suppressor lncRNA MEG3 and its related molecules (miR-9-5p and SOX11) to explore the effects of those interactions on HCC. We measured the expression of MEG3, miR-9-5p, and SOX11 in HCC tissues. Compared to the normal tissues, the results showed that MEG3 and SOX11 were poorly expressed but miR-9-5p was highly expressed in HCC tissues, which is consistent with previous studies (24,26–28). In addition, their expression levels indicated a negative correlation between MEG3 and miR-9-5p and a positive correlation with SOX11.

Several studies suggest that lncRNAs can interact with miRNAs and act as miRNAs sponges to regulate the biological process of cancer (29). lncRNA MEG3 has demonstrated to suppress cell proliferation and induce cell apoptosis, leading to inhibition of tumorigenesis via different regulatory mechanisms (10). MEG3 serves as a ceRNA by sponging miR-183 to regulate the cell growth of pancreatic neuroendocrine tumor (30). Additionally, the interaction between MEG3 and miR-9-5p has been found in prostate cancer, and MEG suppresses prostate cancer progression via mediating miR-9-5p (14). Consistent with previous reports, our study revealed that MEG3 could combine with miR-9-5p and serve as a sponge for miR-9-5p. MEG3 negatively mediated miR-9-5p expression in HCC cells.

miRNAs regulate target gene expression to mediate almost all key biological processes, e.g., cell differentiation, apoptosis, and proliferation (31). Recently, studies have shown that miR-9-5p is relevant to the progression of several cancers (32). Li et al. (33) have found that miR-9-5p can promote lung cancer cell proliferation and migration via suppressing the target gene TGFBR2 expression. Wang et al. have suggested that miR-9-5p mediates GOT1 to suppress cell proliferation, invasion, and glutamine metabolism in pancreatic cancer (34). In this study, the results showed that miR-9-5p was highly expressed in HCC tissues and cells. Moreover, we used StarBase and mirBase software to predict that SOX11 was a direct target of miR-9-5p. The targeting relationship between SOX11 and miR-9-5p was further confirmed by luciferase reporter assay, and this relationship was found for the first time. Furthermore, the data demonstrated that miR-9-5p could negatively mediate SOX11 expression in HCC cells.

The interactions among lncRNA, miRNA, and target gene involved with the pathogenesis of cancers are receiving more attention (35). For instance, lncRNA UCA1 functions as a ceRNA to enhance mitochondrial function and cell viability in bladder cancer by sponging miR-195 to up-regulate ARL2 expression (36). MEG3 can regulate the miR-214/AIFM2 axis to inhibit the growth of lymphoblastic lymphoma (37). Our study further explored the interactions among SOX11, miR-9-5p, and MEG3. The results showed that MEG3 over-expression inhibited cell growth and induced apoptosis through sponging miR-9-5p to enhance SOX11 expression.

In conclusion, this study found that MEG3 was poorly expressed in HCC tissues and cells. Moreover, MEG3 over-expression could regulate the miR-9-5p/SOX11 axis to inhibit cell growth and induce apoptosis in HCC. Therefore, our findings might provide novel molecular mechanisms for understanding the tumorigenesis of HCC and offer potential diagnostic markers and therapeutic targets for HCC.
